# Implementing an enhanced recovery from surgery pathway to reduce hospital length of stay for primary hip or knee arthroplasty: a budget impact analysis

**DOI:** 10.1186/s12913-024-11871-7

**Published:** 2024-12-04

**Authors:** Melanie Lloyd, Zanfina Ademi, Ian A. Harris, Justine Naylor, Peter Lewis, Richard de Steiger, Rachelle Buchbinder, Anthony Wan, Ilana N. Ackerman

**Affiliations:** 1 Centre for Medicine Use and Safety, Monash University, Parkville, Australia; 2 School of Clinical Medicine, Faculty of Medicine and Health, UNSW Sydney, Sydney, Australia; 3 Australian Orthopaedic Association National Joint Replacement Registry, Adelaide, Australia and Faculty of Medicine, University of Adelaide, Adelaide, Australia; 4 Department of Surgery, Epworth HealthCare, University of Melbourne, Melbourne, Australia; 5 School of Public Health and Preventive Medicine, Monash University, Melbourne, Australia; 6 FANZCA, South Western Sydney Clinical School, University of New South Wales, Sydney, Australia; 7 Department of Anaesthetics and Recovery, South Western Sydney Local Health District, Sydney, Australia

**Keywords:** Budget impact analysis, Arthroplasty, Enhanced recovery after surgery, Health service costs

## Abstract

**Background:**

Given growing demand for hip and knee arthroplasty and unsustainable resource requirements, safe and efficient models of care are critical. This study aims to determine the impact on healthcare costs of implementing an enhanced short-stay model of care (ESS-MOC) for arthroplasty at a national level.

**Methods:**

A budget impact analysis was conducted for the years 2023–2030 in the setting of Australian publicly and privately funded hospitals performing hip or knee arthroplasty. The model considered population-based future arthroplasty projections, published data on healthcare costs and resource utilisation, and aggregate health insurer claims data related to minor complexity elective hip or knee arthroplasty for osteoarthritis. The ESS-MOC assigned a conservative hypothesized 30% of eligible patients to an enhanced recovery from surgery (ERAS) pathway which comprised a shortened acute ward stay (average 2 days versus 4 days with current care) and outpatient rehabilitation. The primary outcome was total healthcare cost savings post-ESS-MOC implementation, stratified by joint (knee/hip) and healthcare sector (public/private). Return on investment (ROI) ratio, measuring the return for each dollar invested in implementation, and hospital bed days utilized, were also estimated. Costs are presented in Australian dollars (AUD), at 2023 prices.

**Results:**

Estimated cost savings for 2023–2030 from implementing the ESS-MOC pathway were AUD641 million (95% CI: AUD99 million to AUD1250 million), corresponding to a ROI ratio of AUD8.88 (AUD1.3 to AUD17.9). Total implementation costs for the ESS-MOC were estimated at AUD38 million and AUD34 million for the private and public sectors, respectively. Savings would be 8-fold higher in the private sector (AUD571 million vs. AUD70 million in the public sector), primarily attributable to the > 80,000 rehabilitation bed days saved annually in this sector. For the period 2023–2030, an estimated 337,000 (261,000 to 412,000) acute bed days could be saved (private sector 262,000 [200,000 to 324,000]; public sector 74,000 [57,000 to 92,000]). Less than 10% of eligible patients would need to move into the ERAS pathway to realise cost savings.

**Conclusions:**

Implementation of an enhanced short-stay model of care for eligible arthroplasty patients in Australia would generate significant cost and resource savings, particularly for the private hospital sector.

**Supplementary Information:**

The online version contains supplementary material available at 10.1186/s12913-024-11871-7.

## Background

Internationally, the use of arthroplasty procedures has been rising markedly over time [1]. In Organisation for Economic Co-operation and Development (OECD) countries, average rates of hip arthroplasty have increased from 154 procedures per 100,000 population in 2011 to 179 procedures per 100,000 population in 2019; average rates of knee replacement increased from 109 to 138 procedures per 100,000 population over the same period [2]. In Australia specifically, an estimated AUD3.9 billion is spent on osteoarthritis care annually, with most of the health system expenditure related to hip and knee arthroplasties [3]. Over 52,000 hip and 68,000 knee arthroplasties are performed in Australia each year [4], with surgery volume increasing steadily prior to the COVID-19 pandemic [5, 6]. Modelling based on recent trends and anticipated population growth and ageing has indicated that the number of hip and knee arthroplasties in Australia could rise by 208% and 276%, respectively, by the year 2030 [7]. As with other countries, restrictions on elective (non-urgent) surgery in 2020–2021 due to the COVID-19 pandemic have had major impacts on arthroplasty provision. The Australian Orthopaedic Association National Joint Replacement Registry (AOANJRR) reported that 19,595 fewer arthroplasties were performed during this period [4]. The ‘catch-up’ of unmet need for arthroplasty plus expected rising demand for surgery will present a significant issue for years to come. In this context, safe and efficient models of arthroplasty care will be critical.

Australia has parallel public and private hospital systems. The public system is available to all Australians through the taxpayer-funded Medicare program and access to the private services is predominantly via private health insurance. Around 70% of arthroplasties are undertaken in the private sector [4], where there are minimal waiting lists for elective surgery compared to the public sector. Within the private sector, around half of patients have a post-operative stay in an inpatient rehabilitation facility [8], and this proportion has increased over the past decade [9]. This is despite evidence of comparable clinical outcomes from home-based rehabilitation for most patients [10]. There is growing recognition that rapid recovery and short stay models of care are vital strategies for reducing the cost of arthroplasties in Australia [11]. Enhanced Recovery from Surgery (ERAS) clinical pathways are being implemented for a variety of elective surgical procedures to reduce length of hospital stay. These protocols utilize pre-, intra- and post-operative strategies to enable very early mobilization, early oral feeding, and rapid discharge from hospital [12, 13]. Systematic reviews have found implementation of ERAS pathways for hip and knee arthroplasty can significantly reduce length of hospital stay, without evidence of increased re-admission or common complications [14, 15]. These pathways are commonly limited to patients without extensive comorbidities, those at lower risk of post-operative complications, and those with available supports at home.

While robust economic evaluations of ERAS protocols in arthroplasty are limited, modelling conducted internationally and for other surgical procedures (e.g., colorectal and urologic surgery) suggests that significant health cost savings can be achieved [16, 17]. However, these hinge on the average length of stay (LOS) reduction that can be achieved across all patients, and therefore a minimum level of eligibility and participation must be achieved for the program to make economic sense [17, 18]. Importantly, when determining the economic viability of implementing standardized ERAS protocols for arthroplasty, a comprehensive set of patient population, service provision, and implementation factors should be considered; these have been overlooked in previous evaluations. Budget impact analysis (BIA) evaluates the impact of implementing a novel intervention, model of care or technology on healthcare resource use and the resulting changes in expenditure within a healthcare system [19]. These stand-alone analyses are increasingly undertaken given their importance in reimbursement decisions and health service planning. A BIA forecasts changes in health costs in the short-to-medium term (e.g. future 5–10 years) and can also be used to estimate the projected use of specific resources within a health system, such as hospital bed days or specialist medical appointments when an intervention is adopted at scale, compared to current usual care. This study aimed to evaluate the budget impact of routine implementation of ERAS pathways for appropriate hip or knee arthroplasty patients at a population level, based on a series of conservative and achievable implementation goals.

## Methods

### Design

**Fig. 1:**
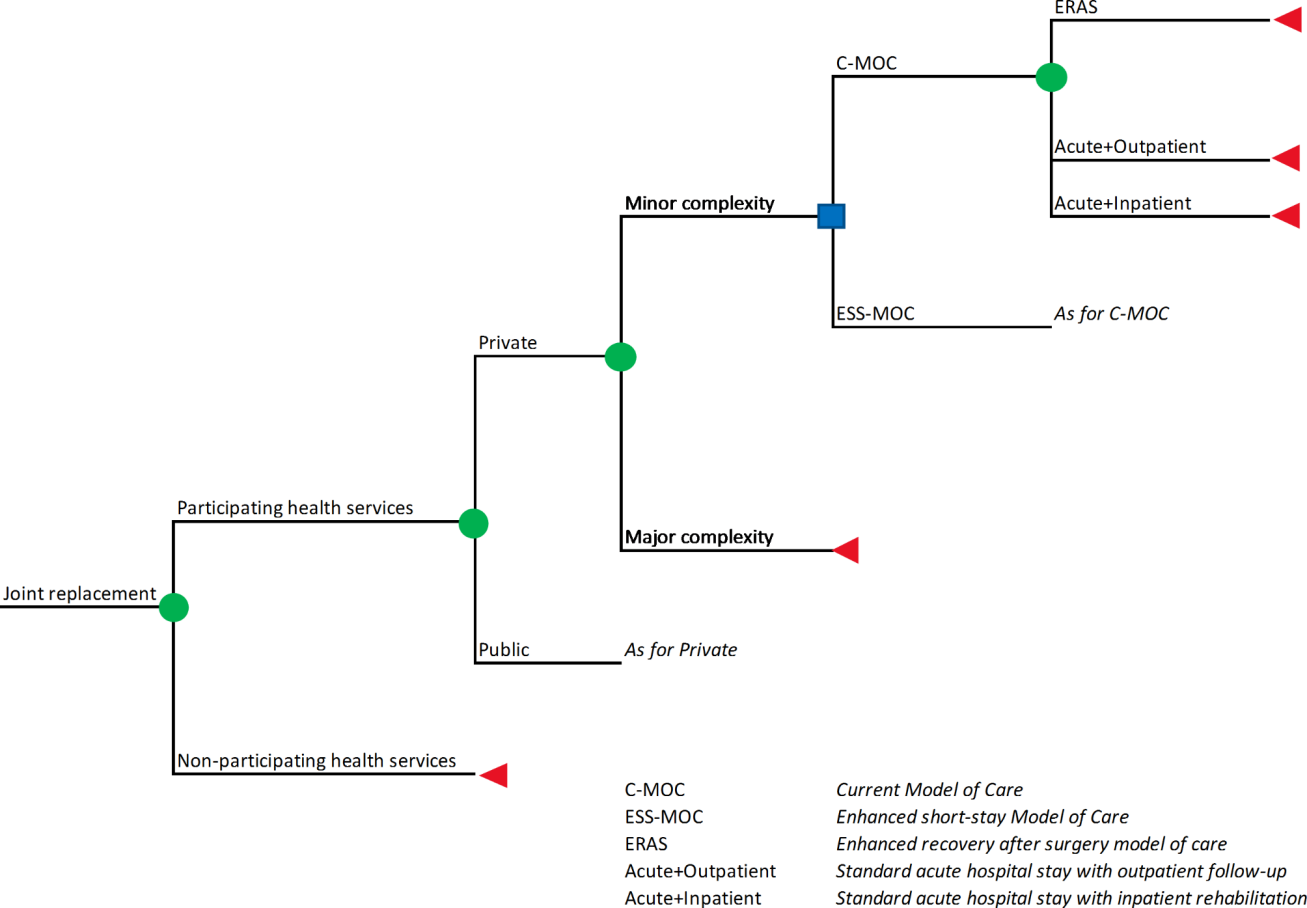
Decision model used in budget impact analysis. Green circles denote a chance node, where a subsequent outcome is based on the probability of an event occurring. Blue squares denote a decision node, where a decision is made between two models of care. Red triangles denote the end of a decision branch. Probabilities assigned to branches are listed in eFigure1 (knee) and eFigure2 (hip)

A budget impact analysis (BIA) was undertaken for non-traumatic primary hip and knee arthroplasty in Australia using a decision-tree model comparing an “enhanced short-stay” model of care (ESS-MOC) with the current model of care (C-MOC) (Fig. 1). Under both models, patients may be assigned to one of three clinical pathways: (1) Short acute hospital stay and outpatient rehabilitation (‘ERAS’); (2) Standard acute hospital stay (as per current routine practice) followed by a period of outpatient rehabilitation (termed ‘Acute + Outpatient’ for this study); or (3) Standard acute hospital stay followed by inpatient rehabilitation on a sub-acute hospital ward (termed ‘Acute + Inpatient’ for this study). Under the ESS-MOC, an increased proportion of patients would receive the ERAS pathway, while the proportion of patients allocated to each pathway under C-MOC is consistent with current practice in Australian hospitals. These proportions are summarized in Table 1. Briefly, acute hospital admission with outpatient rehabilitation is the most common pathway for knee and hip arthroplasty in the private (56% and 60% of procedures, respectively) and public sectors (85% for each). Acute hospital admission with inpatient rehabilitation is more common in the private sector (39% and 33% of knee and hip procedures, respectively) than the public sector (8% and 4% of knee and hip procedures, respectively). Based on available data, the ERAS pathway is currently used infrequently in both sectors (up to 11% of hip or knee arthroplasty procedures in the private and public sectors). The three clinical pathways are outlined in more detail below, and in Fig. 1, eFigure 1 and eFigure 2.

**Table 1:** Proportion of patients allocated to each care pathway under current and enhanced short-stay models of care

Model of care and joint		Private sector			Public sector		
		ERAS	Acute+ Outpatient	Acute+ Inpatient	ERAS	Acute+ Outpatient	Acute+ Inpatient
C-MOC	Knee	0.053	0.557	0.390	0.071	0.854	0.075
	Hip	0.075	0.596	0.329	0.106	0.850	0.044
ESS-MOC	Knee	0.353	0.407	0.240	0.371	0.554	0.075
	Hip	0.375	0.446	0.179	0.406	0.550	0.044

*C-MOC* current model of care, *ESS-MOC* enhanced short-stay model of care, *ERAS* enhanced recovery after surgery

Pathway costs were applied at the population level and extrapolated over 8-years (2023–2030) to estimate the budget impact of a nationwide roll-out of the ESS-MOC to 70% of hospitals, allowing for a proportion of failed uptake by health services. Due to unavailability of data, this proportion of uptake was a conservative assumption that was defined in consultation with the multidisciplinary investigator team, recognising that perfect uptake of a novel model of care is unlikely. As some countries (e.g. Sweden) have implemented short-stay arthroplasty programs in most of their hospitals [20], uptake in 70% of hospitals was assumed to be conservative and achievable for a potentially cost-saving intervention. The 8-year time horizon was chosen to align with the remaining years of published arthroplasty projections for Australia [7]. A healthcare perspective was taken, with consideration of direct costs to the healthcare system. The analysis and results were stratified according to joint (hip / knee) and insurance status (publicly funded / private insurance).

The BIA was conducted and reported in accordance with published guidelines, without discounting applied [19, 21]. Results are presented in Australian dollars (AUD). On 13 September 2023, AUD1.00 was equivalent to US$0.64, UK£0.51, and €0.60 (International Monetary Fund).

### Population

Data for the Australian population receiving elective hip or knee arthroplasty (i.e., non-trauma indication) were obtained from the AOANJRR, which has collected data on 99% of hip and knee arthroplasties performed in Australia since 2002 (> 1.8 million procedures) [4]. In the base case, the annual rate of arthroplasties was projected to grow at a constant rate for the years 2023–2030 (eTable 1a), using national-level projections from a previous study [7]. Based on AOANJRR data for previous years [4], we assumed 70% of procedures would be undertaken in private hospitals for all years of the model. Patients ineligible to receive the ERAS pathway due to severity of comorbidities, complications experienced acutely, functional limitations, or insufficient home supports were excluded from the model (eTable 2).

### Clinical pathways

In Australia, clinical management after hip or knee arthroplasty involves an inpatient stay on an acute surgical ward, with the patient then either discharged directly home or to an inpatient rehabilitation ward. If discharged directly home, the patient may receive either home-based or outpatient rehabilitation (where the patient attends a clinic or hospital for review and treatment), as clinically indicated. If discharged to an inpatient rehabilitation ward, the patient will stay overnight in the ward and continue to receive nursing care alongside onsite rehabilitation sessions. An acute surgical ward stay of less than 2 days with discharge directly home (with either home-based or outpatient rehabilitation) was defined as the ‘ERAS’ pathway. An acute surgical ward stay of more than 2 days and discharge directly home (with home-based or outpatient rehabilitation) was defined as the ‘Acute + Outpatient’ pathway. An acute surgical ward stay (of any duration) with discharge to an inpatient rehabilitation facility was defined as the ‘Acute + Inpatient’ pathway. The current proportion of minor complexity hip and knee arthroplasties assigned to each of these three pathways is listed in Table 1.

In Australian public hospitals, the current average acute surgical ward LOS for a minor complexity arthroplasty is 3.9 days (knee) and 3.7 days (hip) [22]. The average LOS for an inpatient orthopaedic rehabilitation admission is 9 days [22]. In Australian private hospitals, the average LOS is 4.7 days (knee) and 4.4 days (hip) [23]. Based on private health insurer data, the average LOS for private inpatient rehabilitation is 14.7 days (knee) and 13 days (hip). Under current standard care, the average number of outpatient rehabilitation sessions (delivered in the home or at an outpatient rehabilitation setting) varied by hospital sector and by joint (eTables 3 and 4), and was derived from an Australian study of routine clinical practice [8].

A LOS of 2 days was selected as the ERAS pathway target based on prior Australian studies of arthroplasty [24–26]. A comparison of standard ERAS pathway components (as defined in the ERAS Society consensus statement for perioperative hip and knee arthroplasty care [27]), compared to current standard care observed in several tertiary Australian hospitals (as defined by the multidisciplinary expert investigator group) is presented in Table 2). In the base case, we assumed there was no increase in cost or resource use intensity associated with the ERAS protocol pre-, intra- or post-operatively, to accelerate discharge. Instead, resources are redistributed, e.g. a physiotherapy review on the day of surgery rather than Day 1 after surgery and outpatient follow-up commenced earlier but with no increase in the total number of appointments required. This assumption was then tested through a series of scenario analyses, as outlined below, where additional resources are provided to ERAS patients to explore the impact on costs [8].

**Table 2:** Components of the enhanced recovery after surgery (ERAS) protocol

Component	Incorporated into current practice in many Australian hospitals?	Additional cost expected for patients on Enhanced Recovery After Surgery pathway compared to current practice?	Cost (Australian dollars)
Preoperative information, education and counselling	Yes. Short review by anaesthetist and orthopaedic surgeon only; allied health rarely involved unless strong indication.	Base case analysis – No. Scenario 6 – include a structured review with anaesthetics, nursing and allied health.	$541 [22]
Pre-operative optimization (smoking and alcohol cessation, anaemia management, metabolic management, opioid tapering)	Not routinely.	Yes. Outside scope of this model (limited data available, surgical timeframes likely to limit long-term optimization programs). BMI reduction and chronic pain programs likely to be costly.	n/a
Liberal pre-operative fasting protocols (clear fluids up to 2-hours pre-operatively)	Varies but often 6-hour rule applied.	No. Assume absorption of costs within existing operating practices.	n/a
Carbohydrate loading	No	Base case analysis – No. Scenario 7 – Yes. Include carbohydrate drink.	$1^
Standard anaesthetics protocol (local anaesthetic with minimization of opioid use)	Varies – depends on surgeon / anaesthetist preference.	No. Limited data available. Assume absorption of costs within existing operating practices.	n/a
Prophylactic antiemetics. Routine antiemetic started intra-operatively, with regular dose given in first 24 hours.	No	Base case analysis – No. Scenario 7 - Yes. Include pharmaceuticals. Intra-op: - Dexamethasone 8 mg = $1.69 - Ondansetron 4 mg = $0.32 Post-op: − 3 doses of ondansetron 4 mg − 3 x $0.32 = $0.96 At discharge: Ondansetron (oral 4 pack) = $16.^	$19^
Blood conservation	Yes	No change	n/a
Maintain normothermia	Yes	No change	n/a
Judicious IV fluid administration and early oral hydration	No	Yes. Assume absorption of costs within existing operating practices.	n/a
Oral multi-modal analgesia postoperatively	PCA still used in some settings	Yes – oral paracetamol, NSAID. Likely to be cost saving compared to PCA. Assume absorption of costs within existing operating practices.	n/a
Antithrombotic prophylaxis	Yes	No change	n/a
Antimicrobial prophylaxis	Yes	No change	n/a
Very early mobilisation (Day 0, evening)	No	Base case analysis – No. Scenario 8 – include one additional PT session.	$202 (eTable 8)
Early oral feeding	No	Yes, no extra cost.	n/a
Standardized discharge criteria	Varies.	Yes, no extra cost.	n/a
GP visits	0 (only if indicated, not routine).	Base case analysis – No. Scenario 9 – one visit per ERAS patient post-discharge	$77
Outpatient rehab sessions (mean number per patient) – includes outpatient facility and domiciliary sessions (eTable 3).	*Acute + Outpatient* Public hospital: knee = 4.6; hip = 2.6 Private hospital: knee = 2.4; hip = 1.3 *Acute + Inpatient* Public hospital: knee = 4.3; hip = 1.9 Private hospital: knee = 1.4; hip = 1.0	Base case analysis – No. Scenario 10 - Two extra sessions per ERAS patient.	Per session Public: $207 [22] Private: $168 (knee); $164 (hip)

ERAS components adapted from the Consensus statement for hip and knee arthroplasty of the Enhanced Recovery after Surgery (ERAS) Society [27] and the Reporting on ERAS Compliance, Outcomes, and Elements Research (RECOvER) Checklist [28]. Australian clinical experts (orthopaedics, anaesthetics, physiotherapy) were consulted regarding current standard practice

*BMI* body mass index, *ERAS* Enhanced Recovery after Surgery pathway, *GP* General Practitioner, *IV* intravenous, *n/a* not applicable, *NSAID* Non-steroidal anti-inflammatory drug, *PCA* patient controlled analgesia, *PT* Physiotherapy

^While selection of prophylactic medications is likely to vary between hospitals, prices presented here were sourced from the pharmacy of a metropolitan tertiary hospital in Melbourne, Australia. The price for antiemetic drugs dispensed at discharge was taken from the Pharmaceutical Benefits Scheme (www.pbs.gov.au) – Item 5470X. Prices negotiated and paid by individual hospital dispensaries may differ, though we expect little variation in prices for generic medications. Newer antiemetics may attract a higher price

### Effect of selected pathway on patient outcomes

We assumed patient outcomes (complications, procedure-related morbidity and hospital readmissions) would be equivalent, irrespective of pathway. This is consistent with the findings of systematic reviews that have investigated the safety and efficacy of ERAS pathways in appropriately selected patient cohorts [14, 15].

### Patient pathways post-implementation

The allocation of patient pathways under the C-MOC and ESS-MOC is listed in Table 1. Even within the eligible population (based on minor complexity procedures), not all patients will be appropriate for (or willing to accept) a short inpatient stay. For this reason, and consistent with other recent analyses [18], the base case assumed a conservative 30% of eligible arthroplasty patients moved into the ERAS pathway in both the public and private sectors. In the public sector, this 30% was taken from patients receiving the Acute + Outpatient pathway, as strict public hospital eligibility criteria for inpatient rehabilitation suggests a strong indication for remaining in hospital (e.g., lack of social supports at home, significant functional or cognitive limitations). In the private sector, 15% of ERAS pathway patients came from each of the Acute + Outpatient and Acute + Inpatient pathways given evidence suggests that many inpatient rehabilitation admissions in the private sector are based on patient and/or surgeon preference rather than clinical need [9, 29].

### Healthcare costs

Healthcare cost savings refer to the difference (reduction) in costs incurred by the health service for provision of arthroplasty procedures after implementation of the ESS-MOC. For the public sector, the average cost of a minor complexity arthroplasty procedure (including acute surgical ward stay) was AUD20,779 (knee) and AUD21,439 (hip), taken from the National Hospital Cost Data Collection (NHCDC; Round 24 2019-20) [22]. The cost of an ERAS short-stay admission was derived from National Health Performance analysis of joint arthroplasty costs according to LOS (eTables 5 and 6) [30]. The cost of an inpatient rehabilitation admission was AUD9978 (code: AN-SNAP 4A21) [22]. The cost of an outpatient rehabilitation session was AUD207 (code: Tier 2 4009) [22].

Costs of arthroplasty in the private sector were obtained from the insurance claims database of a large private health insurer (Health Fund 1, eTable 4). Average cost of a minor complexity arthroplasty (including acute surgical ward stay) was AUD22,448 (knee) and AUD25,551 (hip). The cost of an ERAS short-stay admission was derived by modelling procedure cost according to LOS (eTables 5 and 6). The cost of an inpatient rehabilitation stay in the private sector was AUD10,557 (knee) and AUD9735 (hip). The estimated cost of an outpatient rehabilitation session was AUD168 (knee) and AUD164 (hip). Healthcare cost parameters and distributions are listed in eTables 4–7, with costs inflated to 2023 prices using the Australian Health Price Index [31].

### Implementation costs

Implementation costs refers to the costs associated with implementing the new model of care and includes staff education and training, audit and monitoring of protocol adherence and purchase of new equipment. In Australia, 170 private and 150 public hospitals offer arthroplasty surgery [4] and each hospital was assumed to incur an implementation cost for the ESS-MOC. The implementation framework and costs are presented in Table 3 and eTables 7, 8 and 9; these were based on prior successful ERAS and Australian hospital clinical pathway implementation models [17, 32]. Key elements included staff training, education materials, employment of project officers and clinical champions, and ongoing audit and feedback. A fixed allowance was provided for capital upgrades required to provide the ERAS pathway [17, 25, 27]. Implementation costs were estimated at AUD172,756 per hospital in year 1, AUD23,036 in year 2 and AUD21,036 for Year 3 onwards (Table 3).

**Table 3:** Average per-hospital resources and costs required for implementation of the enhanced short-stay model of care

Component	Salary in Australian dollars	Per hospital	Number of years cost accrued
Set up and training:			
Training course (Australian dollars)	n/a	10,000	1
Capital expenses (Australian dollars)	n/a	10,000	1
Education materials (Australian dollars)	n/a	2,000	2
Implementation project manager	105,178	0.5 FTE	1
Surgeon leader	292,162	0.1 FTE	1
Anaesthetist leader	292,162	0.1 FTE	1
Nursing leader	93,492	0.2 FTE	1
Management, audit and monitoring:			
Program manager	105,178	0.2 FTE	Ongoing
Total implementation cost per hospital in Australian dollars – Base case			
Year 1			172,756
Year 2			23,036
Ongoing			21,036

Derived from Stone et al. 2016 [17] and Curtis et al. 2021 [32] and adjusted to the Australian arthroplasty context; salary costs include 15% leave loading

*FTE* Full-time equivalent employees, *n/a* not applicable

### Data analysis

Decision tree analysis (Fig. 1, eFigure 1 and eFigure 2) was used to assign healthcare resource use and healthcare costs if the eligible arthroplasty population was managed under: (1) the C-MOC; and (2) the ESS-MOC. The aggregate cost (and mean cost per patient) of minor complexity elective arthroplasties in Australian hospitals implementing the ESS-MOC, compared to the cost under current practice (C-MOC), was calculated for the years 2023–2030. The ROI ratio was calculated as: [(healthcare cost savings – implementation costs) / implementation costs]. Costs are presented in 2023 prices. Analyses were completed in Microsoft Excel with @RISK Analysis Add-in Industrial Edition 8.1.1 (Microsoft Corporation, Redmond, USA).

### Sensitivity and scenario analysis

The base case analysis generated results from running the decision model with the preferred set of assumptions and input values as outlined above. Comprehensive sensitivity and scenario analyses were then conducted to test the impact of varying the assumptions (within plausible ranges) and published input values within given uncertainty intervals, as described below. Deterministic sensitivity analyses were conducted to explore the impact of individual key parameters in the model (healthcare and implementation costs (95% confidence interval (CI) if available or assuming standard error 10% of mean), the proportion of hospitals implementing the ERAS pathway (range +/-25% of mean), proportion of arthroplasty patients moved into the ERAS pathway under the ESS-MOC (range +/-25% of mean)). Probabilistic sensitivity analyses (10,000 iterations) were conducted for the base case to estimate the impact of uncertainty around the parameters listed above, with distributions listed in eTable 7.

A series of scenario analyses was conducted to explore the impact of varying the assumptions underpinning the base case model. This was particularly important where base case parameters were selected based on expert opinion, due to the absence of published evidence. Scenario 1 limited the budget impact analysis to a shorter 5-year timeframe (2023–2027). Scenario 2 utilized alternative arthroplasty rates for the Australian population based on projected exponential growth (eTable 1b) from a previous national-level projections study [7]. Scenario 3 increased the target LOS for the ERAS pathway to 3-days. In Scenario 4, the cost of ERAS implementation was varied from 0 to 200% of the base case implementation cost. Scenario 5 varied the proportion of arthroplasty patients moved into the ERAS pathway under the ESS-MOC between 5% and 50%.

Scenarios 6–10 varied the ongoing healthcare resources required to deliver the ERAS pathway in addition to those available under usual care (Table 2). Scenario 6 included an additional multidisciplinary pre-operative outpatient visit (AUD541, Tier 2 service code: 2002) [22]. Scenario 7 included an increased cost for intra-operative consumables and pharmaceuticals (AUD20). Scenario 8 included an additional early mobilization session with a physiotherapist on the day of surgery (AUD202). Scenario 9 included an additional general medical practitioner visit post-discharge (AUD77, Medical Benefits Scheme Item 36) [33]. Scenario 10 included two additional outpatient rehabilitation sessions, costed as previously described. Scenario 11 combined all these additional care components into a single model. Finally, Scenario 12 utilized healthcare cost parameters for joint arthroplasty from the 2019-20 claims data of an alternative private health funder. While the base case is modelled on claims data provided by Health Fund 1, Scenario 12 used data from Health Fund 2 (eTable 4). Between them, Health Funds 1 and 2 reimburse almost one-third of arthroplasties undertaken in Australian private hospitals.

## Results

### Base case

The ESS-MOC pathway was estimated to produce savings of AUD44 million in 2023, increasing to savings of AUD91 million by 2030 (Table 4). The total savings for the 2023–2030 period were estimated at AUD641 million (95% CI: AUD99 million, AUD1250 million), which includes private sector savings of AUD571 million (AUD55 million to AUD1147 million) and public sector savings of AUD70 million (AUD-94 million to AUD248 million). This corresponded to an overall ROI for the years 2023–2030 of AUD8.88 (AUD1.3 to AUD17.9) for every dollar spent. The ROI would be AUD14.9 (AUD1.3 to AUD30.5) and AUD2.08 (AUD-2.7 to AUD7.4) in the private and public sectors, respectively. This includes implementation costs of AUD38 million for the private and AUD34 million for the public sector.

**Table 4:** Budget impact analysis (Australian dollars in 2023 prices) - base case

	2023	2024	2025	2026	2027	2028	2029	2030	Total 2023–2030 (95% CI)
Total budget savings (in millions)									
Overall	44	79	82	84	85	87	89	91	641 (99, 1250)
Private	50	69	71	73	74	76	78	79	571 (55, 1147)
Public	-6	10	10	11	11	11	11	12	70 (-94, 248)
Per joint									
Overall	791	1,401	1,411	1,413	1,415	1,416	1,418	1,419	
Private	1,261	1,714	1,721	1,723	1,724	1,725	1,726	1,727	
Public	-389	616	632	635	637	640	642	644	
Knee	870	1,480	1,490	1,492	1,494	1,495	1,497	1,498	
Hip	659	1,270	1,279	1,281	1,283	1,284	1,286	1,287	
Return on investment ratio									
Overall									8.9 (1.3, 17.9)
Private									14.9 (1.3, 30.5)
Public									2.1 (-2.7, 7.4)

For the period 2023–2030, it was estimated that a total of 337,000 (261,000 to 412,000) acute bed days could be saved (private sector 262,000 [200,000 to 324,000]; public sector 74,000 [57,000 to 92,000]) (Table 5). Over the same period, it was estimated that a total of 721,000 (471,000 to 1,028,000) rehabilitation bed days could be saved, all within the private sector. Assuming available operating capacity, these bed day savings could create capacity to facilitate an extra 100,000 arthroplasty procedures by 2030 (Table 6).

**Table 5:** Estimated acute and rehabilitation bed day savings from 2023–2030

	2023	2024	2025	2026	2027	2028	2029	2030	Total 2023–2030 (95% CI)
Acute bed days									
Overall (private and public)	38,866	39,808	40,730	41,671	42,550	43,436	44,303	45,141	336,504 (260,781; 412,203)
Knee	25,230	25,839	26,433	27,038	27,596	28,160	28,713	29,247	
Hip	13,635	13,968	14,297	14,633	14,954	15,276	15,590	15,894	
Private – overall	30,266	31,000	31,718	32,451	33,135	33,825	34,500	35,153	262,046 (199,807; 324,277)
Knee	19,611	20,084	20,545	21,016	21,449	21,888	22,318	22,733	
Hip	10,655	10,915	11,172	11,435	11,686	11,937	12,182	12,420	
Public – overall	8,600	8,808	9,012	9,221	9,415	9,611	9,803	9,988	74,458 (56,773; 92,140)
Knee	5,620	5,755	5,888	6,022	6,147	6,272	6,395	6,514	
Hip	2,980	3,053	3,125	3,198	3,268	3,339	3,407	3,474	
Bed days per arthroplasty (all years)									
Overall									0.70
Private									0.77
Public									0.55
Rehabilitation bed days									
Overall (private and public)	83,249	85,268	87,243	89,258	91,140	93,038	94,894	96,689	720,779 (470,551; 1,028,127)
Knee	54,391	55,705	56,984	58,289	59,491	60,707	61,900	63,051	
Hip	28,858	29,563	30,259	30,969	31,649	32,330	32,994	33,638	
Private – overall	83,249	85,268	87,243	89,258	91,140	93,038	94,894	96,689	720,779 (470,551; 1,028,127)
Knee	54,391	55,705	56,984	58,289	59,491	60,707	61,900	63,051	
Hip	28,858	29,563	30,259	30,969	31,649	32,330	32,994	33,638	
Public – overall	0	0	0	0	0	0	0	0	0 (0; 0)
Knee	0	0	0	0	0	0	0	0	
Hip	0	0	0	0	0	0	0	0	
Bed days per arthroplasty (all years)									
Overall									1.51
Private									2.11
Public									0.00

*CI* confidence interval

**Table 6:** Potential increased capacity for arthroplasty throughput, assuming bed availability is the current limiting factor

Year	Number of additional procedures	
	Private	Public
2023	8,331	2,751
2024	8,533	2,818
2025	8,730	2,883
2026	8,932	2,950
2027	9,121	3,012
2028	9,311	3,075
2029	9,497	3,136
2030	9,677	3,195
Total	72,130	23,819

### Sensitivity analysis

**Fig. 2:**
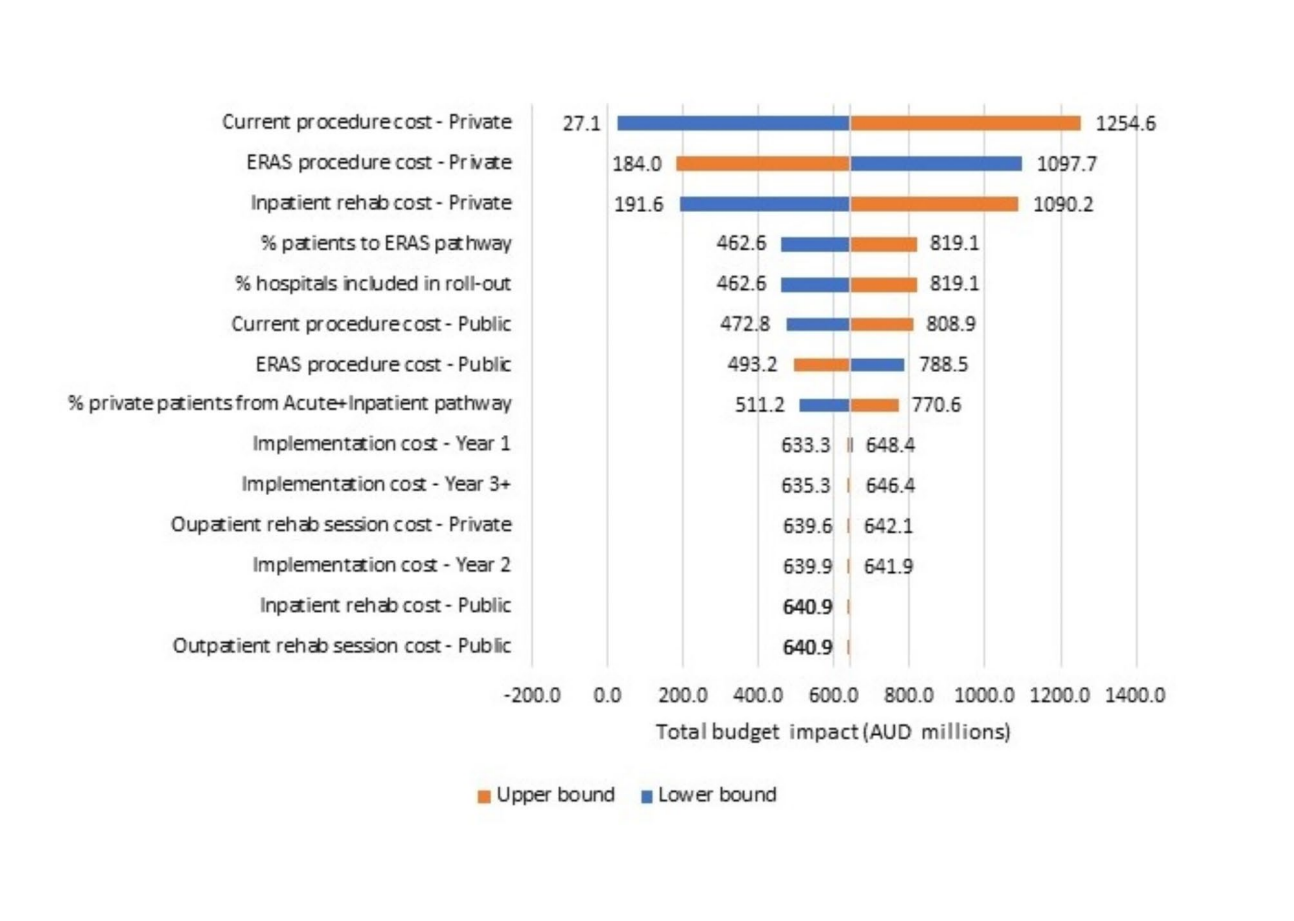
Tornado graph summarizing the deterministic sensitivity analysis for the base case. AUD: Australian dollars; ERAS: Enhanced recovery from surgery pathway

One-way sensitivity analysis showed that private hospital costs (cost of current acute admission, cost of ERAS admission and cost of inpatient rehabilitation admission) contributed most to uncertainty in the model (Fig. 2), however, the model remained cost saving over all parameter ranges explored. Probabilistic sensitivity analyses showed that the probability of the ESS-MOC program being cost saving was 98.6% in the private sector and 79.7% in the public sector.

### Scenario analyses

**Fig. 3:**
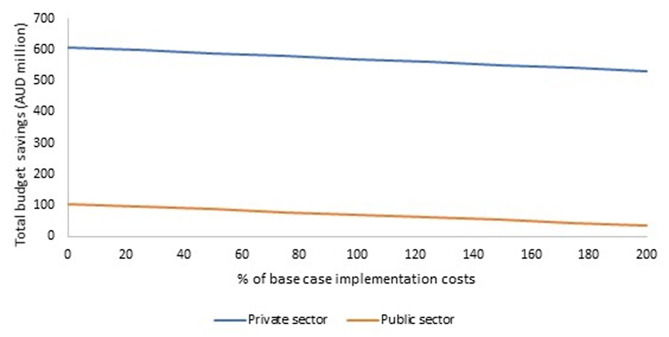
Impact of varying the implementation costs on total budget savings for 2023–2030 (Scenario 4). AUD: Australian dollars

**Fig. 4:**
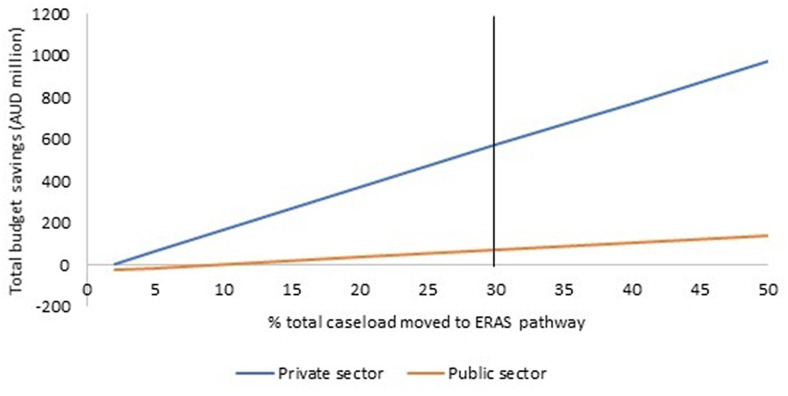
Impact of varying the proportion of patients who are moved into the Enhanced recovery after surgery pathway under ESS-MOC on total budget savings for 2023–2030 (Scenario 5). ERAS: Enhanced recovery from surgery pathway; ESS-MOC: Enhanced short stay model of care. In the public sector, 100% of the ERAS caseload was moved from the Acute+Outpatient pathway; in the private sector, 50% of the ERAS caseload was moved from the Acute+Outpatient pathway and 50% was moved from the Acute+Inpatient pathway. In the base case, 30% of total caseload was moved to the ERAS pathway (represented by the vertical line)

The impact of the scenarios on total health budget and annual expenditure for each year of the program (total and per arthroplasty) is presented in Table 7. The ESS-MOC was cost saving in both the private and public sectors in all scenarios explored. Even if implementation costs are doubled, cost savings of AUD532 million and AUD37 million are projected over the years 2023-30 in the private and public sectors, respectively (Scenario 4, Fig. 3). The ESS-MOC becomes cost saving if more than 2% of eligible private patients and 10% of public patients are transferred to the ERAS pathway (Scenario 5, Fig. 4). The results were robust to alternative sources of private health insurance cost data.

**Table 7:** Results of scenario analyses (Australian dollars in millions, 2023 prices)

		2023	2024	2025	2026	2027	2028	2029	2030	TOTAL (2023 to 2030)	ROI ratio
**Scenario 2**	**Total budget savings**	99	139	154	165	177	189	202	216	**1**,**341**	**18.6**
	**Private**	97	121	132	142	152	163	174	185	**1**,**166**	**30.4**
	**Public**	2	19	21	23	25	26	28	31	**175**	**5.2**
**Scenario 3**	**Total budget savings**	33	68	70	72	74	75	77	79	**549**	**7.6**
	**Private**	46	65	67	69	70	72	73	74	**535**	**14.0**
	**Public**	-13	3	4	4	4	4	4	4	**14**	**0.4**
**Scenario 4**	**See Fig. ****3.**										
**Scenario 5**	**See Fig. ****4.**										
**Scenario 6**	**Total budget savings**	35	70	72	74	76	77	79	81	**563**	**7.8**
	**Private**	43	63	64	66	67	69	70	72	**515**	**13.4**
	**Public**	-9	7	8	8	8	8	9	9	**48**	**1.4**
**Scenario 7**	**Total budget savings**	43	79	81	83	85	87	89	91	**638**	**8.8**
	**Private**	50	69	71	73	74	76	77	79	**568**	**14.8**
	**Public**	-6	10	10	11	11	11	11	12	**69**	**2.1**
**Scenario 8**	**Total budget savings**	40	76	78	80	82	84	85	87	**612**	**8.5**
	**Private**	47	67	69	70	72	73	75	76	**550**	**14.3**
	**Public**	-7	9	9	10	10	10	10	11	**62**	**1.8**
**Scenario 9**	**Total budget savings**	42	78	80	82	84	86	88	89	**630**	**8.7**
	**Private**	49	68	70	72	73	75	77	78	**563**	**14.7**
	**Public**	-6	10	10	10	11	11	11	11	**67**	**2.0**
**Scenario 10**	**Total budget savings**	38	73	75	77	79	81	82	84	**590**	**8.2**
	**Private**	46	65	67	69	70	72	73	75	**536**	**14.0**
	**Public**	-8	8	8	9	9	9	9	9	**53**	**1.6**
**Scenario 11**	**Total budget savings**	24	59	61	62	64	65	67	68	**470**	**6.5**
	**Private**	36	55	57	58	59	61	62	63	**450**	**11.8**
	**Public**	-12	4	4	4	4	5	5	5	**19**	**0.6**
**Scenario 12**	**Total budget savings**	46	82	84	86	88	90	92	94	**663**	**9.2**
	**Private**	52	72	74	76	77	79	81	82	**592**	**15.5**
	**Public**	-6	10	10	11	11	11	11	12	**70**	**2.1**

*ROI* return on investment

## Discussion

National implementation of a short-stay enhanced model of arthroplasty care for 30% of eligible patients would offer significant potential health cost savings and a high ROI for the Australian health system, predominantly for the private hospital sector. The acute bed day savings indicate an important opportunity for increasing surgical throughput, particularly in the context of anticipated rising demand for arthroplasty [7], ‘catch up’ of unmet need due to COVID [4], and growth in the proportion of patients waiting more than 12 months for surgery [34].

Prior economic evaluation of ERAS protocols has produced strong evidence of cost savings for health funders [17]. However, our findings indicate that careful consideration of context (for example, private versus public sector) and the realistic likelihood of achieving meaningful reductions in LOS is necessary. Projected cost savings hinge on being able to move patients away from inpatient rehabilitation to a shorter hospital stay with outpatient or home-based rehabilitation. With few arthroplasty patients currently receiving inpatient rehabilitation in public hospitals, the potential for reduction in public rehabilitation bed days is very limited. In contrast, a large proportion of private hospital patients receive inpatient rehabilitation under the C-MOC, presenting significant opportunity for cost savings through reduced LOS or alternative rehabilitation services. However, patients may need to be incentivized to go home early, potentially through funded home services and support for carer costs.

To support ERAS implementation, there is a need for guidelines tailored to the Australian setting to promote consistency in pre-, peri- and post-operative practices. Validated screening tools to predict clinical need for inpatient rehabilitation and improved pre-operative patient education around discharge destination are also critical [8, 10]. To achieve large increases in ERAS uptake, dedicated pre-operative preparation (e.g., patient education and setting appropriate expectations for the short stay and early discharge) will likely be required and could address some of the identified barriers to accepting home-based care after arthroplasty [29].

While there are economic and resource use advantages from implementing the ERAS pathway, viability also relies on no increase in adverse patient outcomes. It is therefore recommended that large-scale implementation of these programs be accompanied by careful monitoring of post-operative outcomes and key quality indicators (such as readmissions, complications and rates of revision procedures) to confirm comparable ‘real-world’ safety. While not specific to short-stay care models of care, a composite outcome measure for evaluating the quality of arthroplasty care (based on four indicators) has been reported previously [35]. Higher readmission rates, in particular, have the potential to quickly eliminate hospital cost savings. It is also important that care provision is not shifted from hospitals to community-based medical, nursing and allied health services without appropriate resourcing of these services. Improved access to post-discharge services will be needed, particularly for people living in non-metropolitan areas and those receiving arthroplasty in the public system. Shifting inpatient physiotherapy care to outpatient settings may generate substantial out-of-pocket costs which could impact the patient acceptability of the program [8, 10].

Strengths of this study include the use of highly representative national data from the AOANJRR, NHCDC and two large private health insurers which between them fund almost one-third of the private elective arthroplasties provided in Australia annually. Our modelling considered both healthcare sectors, and acute and rehabilitation components. Importantly, we included anticipated implementation costs which are often overlooked in health economic evaluations, but should be considered alongside treatment costs within decision making [36]. We considered the major pre-operative, intra-operative and post-operative model inputs, and performed multiple scenario analyses to quantify uncertainty. The model is a dynamic resource that can be updated as new population, costs, comparative effectiveness and safety data emerge. As we adopted a conservative approach to all cost estimates, potential savings may be underestimated.

The model outcomes rely on several important assumptions. We acknowledge that a true estimate of patient eligibility for the ERAS pathway is difficult given multiple relevant patient factors (including overall health, functional independence, and social supports). While we assumed a conservative 30% of arthroplasty patients from 70% of hospitals nationwide will move to the ERAS pathway, sensitivity analysis showed that the new model would be cost saving even with a very small proportion of patients. Due to difficulty in obtaining reliable cost estimates, impacts on health resource use were not exhaustively explored in scenario analyses. Other potentially relevant costs include domiciliary assistance, nursing and rehabilitation, and equipment. It is likely that some ERAS pathway costs cannot be absorbed through resource re-allocation. Systematic reviews have demonstrated significant variability between ERAS protocols and the optimal model of care remains unclear [14, 15]. ERAS acceptability and feasibility aspects that may impact nationwide implementation could also be important. We adopted a health system perspective that focused on direct costs and did not include costs to patients or carers. We recognise that while indirect costs (for example, time taken off work by carers after early hospital discharge and for patient travel to and from post-discharge care settings) are not considered in our budget impact analysis given the lack of reliable data, they form part of the overall financial impact of short stay implementation.

The generalizability of these results to other countries and health systems is impacted by the degree to which ERAS and short stay models of care are already utilized. While short-stay arthroplasty programs have been implemented to varying degrees in the United States, United Kingdom, and Denmark [14, 15, 17], other international jurisdictions have made little progress in modernizing models of care to improve efficiency and ensure judicious use of health system resources. In particular, an average LOS for arthroplasty greater than 10 days persists in several other European and Asian countries [37]. In settings where uptake has been low, robust economic evidence of the potential value of safe and efficient models of care will likely be critical for driving significant policy and practice change. Our model can be adapted, using local population, healthcare resource use and costs inputs from other jurisdictions, to estimate potential healthcare savings that could be achieved through short-stay implementation in other countries.

## Conclusions

Implementation of an enhanced short-stay model of care for an additional 30% of eligible hip or knee arthroplasty patients in Australia would generate significant cost savings for the health system with a very high ROI. The cost savings would be 8-fold higher for the private sector. Reductions in acute ward bed days would be substantial in both hospital sectors under the new model of care. Our budget impact analysis represents a best practice approach to comprehensively assessing the potential value, at a national level, of implementing new models of care in high-burden healthcare contexts.

## Supplementary Information

Supplementary Material 1.

## Data Availability

All data used in this study has already been published and is publicly available, with links to online sources referenced within the document as relevant. The only data that is not publicly available is that of the private health funders which is confidential for commercial and patient confidentiality reasons. Summary data, as used in the modelling and analysis, is provided in the electronic supplementary materials.

## Abbreviations

AOANJRR: Australian Orthopaedic Association National Joint Replacement Registry
AUD: Australian dollar
BIA: Budget impact analysis
CI: Confidence interval
C-MOC: Current model of care
ERAS: Enhanced recovery after surgery
ESS-MOC: Enhanced short stay model of care
LOS: Length of stay
NHCDC: National hospital cost data collection
