# Supplementary material for: Implementing an enhanced recovery from surgery pathway to reduce hospital length of stay for primary hip or knee arthroplasty: a budget impact analysis
Source: BMC Health Serv Res. 2024 Dec 4;24:1540. doi: 10.1186/s12913-024-11871-7 (PMC11616323; doi:10.1186/s12913-024-11871-7)
Supplement: Supplementary file 1 — Supplementary Material 1. [file 12913_2024_11871_MOESM1_ESM.docx]

**Additional file 1**

**Budget impact of implementing an enhanced recovery from surgery pathway to reduce hospital length of stay for primary hip and knee replacement surgery in Australia**

M Lloyd, Z Ademi, IA Harris, J Naylor, P Lewis, R de Steiger, R Buchbinder, A Wan, IN Ackerman

**Table of contents**

[eTable 1a. Projected number of joint replacements in Australia for 2023-2030 (based on constant growth model) - used in Base Case 3](#_Toc177048839)

[eTable 1b. Projected number of joint replacements in Australia 2023-2030 (based on exponential growth model) - used in Scenario 2 3](#_Toc177048840)

[eTable 2. Proportion of arthroplasty patients excluded from budget impact analysis due to major complexity procedure 4](#_Toc177048841)

[eTable 3. Derivation of average number of outpatient rehabilitation sessions received per patient by joint and pathway for the public hospital sector 5](#_Toc177048842)

[eTable 4. Private health fund cost and resource use data according to procedure code 6](#_Toc177048843)

[eTable 5. Derivation of cost of Enhanced recovery after surgery admission for knee arthroplasty in Australian dollars 8](#_Toc177048844)

[eTable 6. Cost of Enhanced recovery after surgery admission for hip arthroplasty in Australian dollars 9](#_Toc177048845)

[eTable 7. Parameters used in Deterministic and Probabilistic Sensitivity Analysis 10](#_Toc177048846)

[eTable 8. Indicative published Enterprise Bargaining Agreement salaries for public sector health professionals in Australia (2022) 12](#_Toc177048847)

[eTable 9. Number of Australian hospitals performing arthroplasty surgery 13](#_Toc177048848)

[eFigure 1. Decision tree showing probabilities assigned to chance branches for knee arthroplasties 14](#_Toc177048849)

[eFigure 2. Decision tree showing probabilities assigned to chance branches for hip arthroplasties 15](#_Toc177048850)

[Additional file references 16](#_Toc177048851)

## eTable 1a. Projected number of joint replacements in Australia for 2023-2030 (based on constant growth model) - used in Base Case

|  | **2023** | **2024** | **2025** | **2026** | **2027** | **2028** | **2029** | **2030** |
| --- | --- | --- | --- | --- | --- | --- | --- | --- |
| ALL HOSPITALS | | | | | | | | |
| Knee | 56,563 | 57,929 | 59,260 | 60,616 | 61,867 | 63,132 | 64,372 | 65,569 |
| Hip | 33,934 | 34,763 | 35,582 | 36,418 | 37,216 | 38,018 | 38,798 | 39,556 |
| Total | 90,498 | 92,693 | 94,842 | 97,034 | 99,083 | 101,150 | 103,170 | 105,125 |
| PRIVATE HOSPITALS | | | | | | |  |  |
| Knee | 39,594 | 40,551 | 41,482 | 42,431 | 43,307 | 44,192 | 45,061 | 45,898 |
| Hip | 23,754 | 24,334 | 24,907 | 25,492 | 26,051 | 26,613 | 27,159 | 27,689 |
| Total | 63,349 | 64,885 | 66,389 | 67,924 | 69,358 | 70,805 | 72,219 | 73,587 |
| PUBLIC HOSPITALS | | | | | | |  |  |
| Knee | 16,969 | 17,379 | 17,778 | 18,185 | 18,560 | 18,940 | 19,312 | 19,671 |
| Hip | 10,180 | 10,429 | 10,675 | 10,925 | 11,165 | 11,405 | 11,639 | 11,867 |
| Total | 27,149 | 27,808 | 28,453 | 29,110 | 29,725 | 30,345 | 30,951 | 31,537 |

Based on constant growth projections from Ackerman et al 2019^1^

## eTable 1b. Projected number of joint replacements in Australia 2023-2030 (based on exponential growth model) - used in Scenario 2

|  | **2023** | **2024** | **2025** | **2026** | **2027** | **2028** | **2029** | **2030** |
| --- | --- | --- | --- | --- | --- | --- | --- | --- |
| ALL HOSPITALS | | | | | | | | |
| Knee | 98,325 | 102,556 | 113,763 | 122,298 | 131,165 | 140,650 | 150,676 | 161,231 |
| Hip | 52,438 | 55,813 | 59,350 | 63,110 | 66,988 | 71,080 | 75,351 | 79,795 |
| Total | 150,763 | 158,369 | 173,113 | 185,408 | 198,153 | 211,730 | 226,028 | 241,026 |
| PRIVATE HOSPITALS | | | | | | | | |
| Knee | 68,827 | 71,790 | 79,634 | 85,609 | 91,816 | 98,455 | 105,473 | 112,862 |
| Hip | 36,707 | 39,069 | 41,545 | 44,177 | 46,891 | 49,756 | 52,746 | 55,856 |
| Total | 105,534 | 110,858 | 121,179 | 129,786 | 138,707 | 148,211 | 158,219 | 168,718 |
| PUBLIC HOSPITALS | | | | | | |  |  |
| Knee | 29,497 | 30,767 | 34,129 | 36,690 | 39,350 | 42,195 | 45,203 | 48,369 |
| Hip | 15,731 | 16,744 | 17,805 | 18,933 | 20,096 | 21,324 | 22,605 | 23,938 |
| Total | 45,229 | 47,511 | 51,934 | 55,622 | 59,446 | 63,519 | 67,808 | 72,308 |

Based on exponential growth projections from Ackerman et al 2019^1^

## eTable 2. Proportion of arthroplasty patients excluded from budget impact analysis due to major complexity procedure

|  | **Private sector** | **Public sector** |
| --- | --- | --- |
| Knee | 0.11 | 0.17 |
| Hip | 0.11 | 0.18 |

Source: Based on proportion of total arthroplasties assigned a “major complexity” Diagnosis-Related Group admission code (I04A, I33A) in the National Hospital Cost Data Collection Round 24 (2019-20) Public^2^ and Round 23 (2018-19) Private^3^.

## eTable 3. Derivation of average number of outpatient rehabilitation sessions received per patient by joint and pathway for the public hospital sector

|  | **Knee** | | **Hip** | |
| --- | --- | --- | --- | --- |
|  | n | % | n | % |
| Monitored | 2 | 19.2 | 2 | 38.3 |
| Domicillary | 4 | 6.7 | 4.5 | 5.6 |
| Outpatient | 6 | 59.6 | 6 | 24.6 |
| Unmonitored | 0 | 7.1 | 0 | 27 |
|  |  |  |  |  |
| Average sessions per patient |  |  |  |  |
| Average Acute+Outpatient, ERAS pathway | 4.57 |  | 2.61 |  |
| Average Acute+Inpatient pathway^a^ | 4.32 |  | 1.90 |  |

^a^Allowing for 0.4% of knee and 1.2% of hip patients receiving inpatient rehab only

Derived from Naylor et al. (2019)^4^

ERAS: Enhanced recovery after surgery

## eTable 4. Private health fund cost and resource use data according to procedure code

|  | **Knee replacements*** | | **Hip replacements**** | |
| --- | --- | --- | --- | --- |
|  | **Health Fund 1** | **Health Fund 2** | **Health Fund 1** | **Health Fund 2** |
| N | 6,440 | 1,691 | 4,384 | 2,015 |
| Mean age (years) | 68.8 | 68.2 | 68.1 | 68.0 |
| Females (proportion) | 0.558 | 0.530 | 0.547 | 0.530 |
|  |  |  |  |  |
| Mean acute LOS (days) | 4.65 | 4.4 | 4.40 | 4.1 |
| Mean rehab LOS (days) | 14.7 | 10.2 | 13.0 | 10.1 |
| % discharged <= 2 nights | 0.053 | 0.062 | 0.075 | 0.109 |
| Proportion with inpatient rehab admission | 0.390 | 0.383 | 0.329 | 0.321 |
|  |  |  |  |  |
| Acute admission costs in AUD by length of stay (nights) | |  |  |  |
| 1 | 20,264 | 14,851 | 25,249 | 19,597 |
| 2 | 22,717 | 18,638 | 26,679 | 20,911 |
| 3 | 23,146 | 22,710 | 26,831 | 25,589 |
| 4 | 23,829 | 23,745 | 27,082 | 26,306 |
| 5 | 24,200 | 24,868 | 27,487 | 26,758 |
| 6 | 24,661 | 25,684 | 27,762 | 26,985 |
| 7 | 24,850 | 26,759 | 28,460 | 28,476 |
| 8 | 25,432 | 26,936 | 28,147 | 28,877 |
| 9 | 25,650 | 28,308 | 28,971 | 31,715 |
| 10 | 25,508 | 30,265 | 29,543 | 28,881 |
| 11 | 27,315 | 38,505 | 32,591 | 30,723 |
|  |  |  |  |  |
| Mean acute admission cost (AUD) | 22,448 | 23,942 | 25,551 | 25,870 |
| ERAS admission cost (AUD) | 21,514 | 20,750 | 24,765 | 23,565 |
| Mean inpatient rehab cost (AUD) | 10,557 | 7,142 | 9,735 | 7,426 |

|  | **Knee replacements*** | | **Hip replacements*** | |
| --- | --- | --- | --- | --- |
|  | **Health Fund 1** | **Health Fund 2** | **Health Fund 1** | **Health Fund 2** |
| Proportion with day rehabilitation (no inpatient rehabilitation) | 0.075 | 0.083 | 0.062 | 0.052 |
| Mean day rehabilitation sessions^6^ | 8 | 8 | 8 | 8 |
| Mean day rehabilitation cost (AUD) | 3708 | 1360 | 2989 | 1147 |
| Mean cost per session (AUD) | 463.52 | 170.00 | 373.67 | 143.38 |
|  |  |  |  |  |
| Proportion attending private physiotherapy | 0.278 | 0.278 | 0.170 | 0.170 |
| Average number of private PT sessions | 3.1 | 3.1 | 2.2 | 2.2 |
| Cost outpatient physiotherapy sessions (AUD) | 87.6 | 89 | 87.6 | 89 |
|  |  |  |  |  |
| Proportion discharged with unmonitored home program | 0.257 | 0.256 | 0.439 | 0.457 |
|  |  |  |  |  |
| Acute+Outpatient MOC |  |  |  |  |
| Mean number of outpatient rehab sessions for patients NOT attending inpatient rehab | 2.40 | 2.47 | 1.29 | 1.16 |
| Mean cost per session (AUD) | 167.6 | 107.6 | 163.8 | 101.7 |
|  |  |  |  |  |
| Acute+Inpatient MOC |  |  |  |  |
| Proportion attending both inpatient and outpatient rehab | 0.170 | 0.100 | 0.124 | 0.080 |
| Mean number of outpatient rehab sessions for patients ALSO attending inpatient rehab | 1.360 | 0.800 | 0.990 | 0.640 |
| Mean cost per session | 167.6 | 107.6 | 163.8 | 101.7 |

AR-DRG: Australian refined diagnosis-related group; AUD: Australian dollars; ERAS: Enhanced recovery after surgery; LOS: length of stay; MOC: model of care; PT: Physiotherapy

* Australian refined diagnosis-related group AR-DRG 104B

** Australian refined diagnosis-related group AR-DRG 133B

## eTable 5. Derivation of cost of Enhanced recovery after surgery admission for knee arthroplasty in Australian dollars

|  | **PUBLIC^a^** | | **PRIVATE HEALTH FUND1^b^** | **PRIVATE HEALTH FUND 2^b^** |
| --- | --- | --- | --- | --- |
| **Length of stay** | **2012 prices** | **2023 prices** | **2023 prices** | **2023 prices** |
| **0** | 9,200 | 10,989 | - | - |
| **1** | 13,200 | 15,766 | 20,264 | 14,851 |
| **2** | 14,500 | 17,319 | 22,717 | 18,638 |
| **3** | 15,800 | 18,872 | 23,146 | 22,710 |
| **4** | 17,200 | 20,544 | 23,829 | 23,745 |
| **5** | 18,400 | 21,978 | 24,200 | 24,868 |
| **6** | 19,700 | 23,530 | 24,661 | 25,684 |
| **7** | 20,000 | 23,889 | 24,850 | 26,759 |
| **8** | - | - | 25,432 | 26,936 |
| **9** | - | - | 25,650 | 28,308 |
| **10** | - | - | 25,508 | 30,265 |

ERAS: Enhanced recovery after surgery pathway; LOS: length of stay on acute surgical ward (nights)

^a^Source: National Health Performance Authority.^5^

^b^Source: Private health insurance claims databases of two large and independent Australian health insurers (unpublished).

**Notes:**

Line of best fit (Public): Average cost of knee arthroplasty procedure = $14238 + ($1553 x LOS). Model applies over range 2-7 nights only.

Average cost of ERAS admission (Public) = (($1553 x ERAS-LOS + $14238)/($1553 x Acute-LOS + $14238)) x Average cost of Acute admission.

$17768 = (($1553 x 2 + $14238)/($1553 x 3.9 + $14238)) x $20779

Line of best fit (Private 1): Average cost of knee arthroplasty procedure = $22190 + ($376 x LOS). Model applies over range 2-10 nights only.

Line of best fit (Private 2): Average cost of knee arthroplasty procedure = $18166 + ($1193 x LOS). Model applies over range 2-10 nights only.

## eTable 6. Cost of Enhanced recovery after surgery admission for hip arthroplasty in Australian dollars

|  | **PUBLIC** | | **PRIVATE HEALTH FUND 1^b^** | **PRIVATE HEALTH FUND 2^b^** |
| --- | --- | --- | --- | --- |
| **Length of stay** | **2012 prices^a^** | **2023 prices** | **2023 prices** | **2023 prices** |
| 0 | 8,200 | 9,794 | - | - |
| 1 | 12,800 | 15,289 | 25,249 | 19,597 |
| 2 | 15,500 | 18,514 | 26,679 | 20,911 |
| 3 | 16,200 | 19,350 | 26,831 | 25,589 |
| 4 | 16,800 | 20,066 | 27,082 | 26,306 |
| 5 | 18,200 | 21,739 | 27,487 | 26,758 |
| 6 | 18,800 | 22,455 | 27,762 | 26,985 |
| 7 | 19,300 | 23,053 | 28,460 | 28,476 |
| 8 | 20,700 | 24,725 | 28,147 | 28,877 |
| 9 | 21,200 | 25,322 | 28,971 | 31,715 |
| 10 | 21,800 | 26,039 | 29,543 | 28,881 |

ERAS: Enhanced recovery after surgery pathway; LOS: length of stay on acute surgical ward (nights)

^a^Source: National Health Performance Authority.^5^

^b^Source: Private health insurance claims databases of two large and independent Australian health insurers (unpublished).

**Notes:**

Line of best fit (Public): Average cost of hip arthroplasty procedure = $16498 + ($977 x LOS). Model applies over range 2-10 nights only.

Average cost of ERAS admission (Public) = (($977 x ERAS-LOS + $16498)/($977 x Acute-LOS + $16498)) x Average cost of Acute admission.

$19668 = (($977 x 2 + $16498)/($977 x 3.7 + $16498)) x $21439

Line of best fit (Private 1): Average cost of hip arthroplasty procedure = $25787 + ($350 x LOS). Model applies over range 2-10 nights only.

Line of best fit (Private 2): Average cost of hip arthroplasty procedure = $21454 + ($952 x LOS). Model applies over range 2-10 nights only.

## eTable 7. Parameters used in Deterministic and Probabilistic Sensitivity Analysis

| **Parameter** |  | **Distribution** | **Mean** | **Standard error** | **Lower bound** | **Upper bound** | **Source** |
| --- | --- | --- | --- | --- | --- | --- | --- |
| Costs in Australian dollars | | | | | | | |
| Private sector – Knee | ERAS procedure | Gamma | 21,514 | 10% of mean | 16,016 | 23,824 | Derived – See eTable6 |
|  | Current procedure | Gamma | 22,448 | 2908 | 23,690 | 24,194 | Health Fund 1 dataset |
|  | Inpatient rehab | Gamma | 10,557 | 4651 | 6788 | 7,496 | Health Fund 1 dataset |
|  | Outpatient rehab session | Gamma | 168 | 10% of mean | 135 | 200 | Health Fund 1 dataset |
| Private sector – Hip | ERAS procedure | Gamma | 24,765 | 10% of mean | 18,212 | 27,091 | Derived – See eTable6 |
|  | Current procedure | Gamma | 25,550 | 3,298 | 25,649 | 26,091 | Health Fund 1 dataset |
|  | Inpatient rehab | Gamma | 9,735 | 4,175 | 7056 | 7,796 | Health Fund 1 dataset |
|  | Outpatient rehab session | Gamma | 164 | 10% of mean | 132 | 196 | Health Fund 1 dataset |
| Public sector – Knee | ERAS procedure | Gamma | 17,761 | 10% of mean | 14,280 | 21,242 | Derived – See eTable6 |
|  | Current procedure | Gamma | 20,779 | 10% of mean | 16,706 | 24,851 | NHCDC^2^ |
|  | Inpatient rehab | Gamma | 9,978 | 10% of mean | 8023 | 11,934 | NHCDC^2^ |
|  | Outpatient rehab session | Gamma | 207 | 10% of mean | 166 | 247 | NHCDC^2^ |
| Public sector – Hip | ERAS procedure | Gamma | 19,668 | 10% of mean | 15,813 | 23,523 | Derived – See eTable6 |
|  | Current procedure | Gamma | 21,439 | 10% of mean | 17,237 | 25,641 | NHCDC^2^ |
|  | Inpatient rehab | Gamma | 9,978 | 10% of mean | 8023 | 11,934 | NHCDC^2^ |
|  | Outpatient rehab session | Gamma | 207 | 10% of mean | 166 | 247 | NHCDC^2^ |

| **Parameter** |  | **Distribution** | **Mean** | **Standard error** | **Lower bound** | **Upper bound** | **Source** |
| --- | --- | --- | --- | --- | --- | --- | --- |
| Total Implementation cost – Private sector (119 hospitals) | Year 1 | Gamma | 20,557,925 | 10% of mean | 16,528,572 | 24,587,279 | Derived – See eTable 3 |
|  | Year 2 | Gamma | 2,741,245 | 10% of mean | 2,203,961 | 3,278,529 | Derived – See eTable 3 |
|  | Year 3+ | Gamma | 2,503,245 | 10% of mean | 2,012,609 | 2,993,881 | Derived – See eTable 3 |
| Total Implementation cost – Public sector (105 hospitals) | Year 1 | Gamma | 18,139,346 | 10% of mean | 14,584,034 | 21,694,658 | Derived – See eTable 3 |
|  | Year 2 | Gamma | 2,418,746 | 10% of mean | 1,944,672 | 2,892,820 | Derived – See eTable 3 |
|  | Year 3+ | Gamma | 2,208,746 | 10% of mean | 1,775,832 | 2,641,660 | Derived – See eTable 3 |
| Other parameters: | | | | | | | |
| % hospitals included in roll-out | | Uniform +/-25% of mean | 70% | n/a | 52.5% | 87.5% | Assumption |
| % total patients at included hospitals moved into ERAS pathway under ESS-MOC | | Uniform +/-25% of mean | 30% | n/a | 22.5% | 37.5% | Assumption |
| % private patients moved to ERAS from Acute+Inpatient pathway | | Uniform +/-25% of mean | 50% | n/a | 37.5% | 62.5% | Assumption |

ERAS: enhanced recovery after surgery pathway; ESS-MOC: Enhanced short-stay model of care; n/a: not applicable; NHCDC: National Healthcare Cost Data Collection.

## eTable 8. Indicative published Enterprise Bargaining Agreement salaries for public sector health professionals in Australia (2022)

|  | **Victoria** | | **Queensland** | | **New South Wales** | | **Tasmania** | |
| --- | --- | --- | --- | --- | --- | --- | --- | --- |
|  | **Grade** | **Salary** | **Grade** | **Salary** | **Grade** | **Salary** | **Grade** | **Salary** |
| **Physiotherapist** | Level 2, Year 2 | 88,920 | HP3.4 | 91,200 | Level 2, Year 2 | 90,261 | Level 2, Year 2 | 84,431 |
| **Registered Nurse** | RN, Year 4 | 79,196 | Band 5, Step 4 | 88,153 | RN, Year 4 | 78,388 | RN Grade 3, Year 4 | 76,974 |
| **Medical Specialist** | HM37, Year 5 | 298,532 | L27 | 239,377 | Staff specialist (senior) | 241,940 | Senior specialist, level 1 | 234,726 |
| **Selected midpoint full-time equivalent salary (Australian dollars; 2022 prices)** | | | | | | | | |
| **Physiotherapist** | | | | | | |  | 90,000 |
| **Registered Nurse** | | | | | | |  | 80,000 |
| **Medical specialist** | | | | | | |  | 250,000 |

**Notes:**

Calculation of cost of early mobilisation session with physiotherapist:

Number of patients reviewed per day (Monday to Friday) = 10; per year = 520.

Annual salary cost = (assuming 15% loading for leave and inflating to 2023 prices) = $105,178.

Cost per patient review (2023 prices) = $202.

Physiotherapist salary was also applied to the Project Manager role for ERAS pathway implementation.

## eTable 9. Number of Australian hospitals performing arthroplasty surgery

| **State or Territory** | **Private** | **Public** |
| --- | --- | --- |
| Victoria | 45 | 42 |
| New South Wales | 54 | 48 |
| Queensland | 34 | 22 |
| Western Australia | 13 | 12 |
| South Australia | 14 | 19 |
| Tasmania | 5 | 3 |
| Australian Capital Territory | 4 | 2 |
| Northern Territory | 1 | 2 |
| TOTAL | 170 | 150 |
| Total hospitals included in ESS-MOC roll-out (70%) | 119 | 105 |
| Total minor complexity arthroplasties completed in included hospitals (2023) | 39,466 | 15,703 |
| Average minor complexity arthroplasties per hospital (2023) | 332 | 150 |
| Projected number of ERAS patients per hospital per year (base case, 2023) | 126 | 57 |

ERAS: Enhanced recovery after surgery; ESS-MOC: Enhanced short-stay model of care

Source of hospital numbers: Australian Orthopaedic Association National Joint Replacement Registry 2022 annual report^6^

## eFigure 1. Decision tree showing probabilities assigned to chance branches for knee arthroplasties

Green circles denote a chance node, where a subsequent outcome is based on the probability of an event occurring. Blue squares denote a decision node, where a decision is made between two models of care. Red triangles denote the end of a decision branch.

## eFigure 2. Decision tree showing probabilities assigned to chance branches for hip arthroplasties

Green circles denote a chance node, where a subsequent outcome is based on the probability of an event occurring. Blue squares denote a decision node, where a decision is made between two models of care. Red triangles denote the end of a decision branch.

## Additional file references

1. Ackerman IN, Bohensky MA, Zomer E, et al. The projected burden of primary total knee and hip replacement for osteoarthritis in Australia to the year 2030. BMC Musculoskeletal Disorders. 2019;20(1):90.

2. Independent Hospital Pricing Authority. National Hospital Cost Data Collection Report: Public Sector, Round 24 (2019–20). https://www.ihacpa.gov.au/resources/national-hospital-cost-data-collection-nhcdc-public-hospital-report-round-24-financial-year-2019-20. Accessed 31 January 2023.

3. Independent Hospital Pricing Authority. National Hospital Cost Data Collection: Private Hospital Report, Round 23 (2018-19). Canberra: Australian Government; 2021.

4. Naylor JM, Hart A, Harris IA, Lewin AM. Variation in rehabilitation setting after uncomplicated total knee or hip arthroplasty: A call for evidence-based guidelines. BMC Musculoskeletal Disorders. 2019;20(1):214.

5. National Health Performance Authority. Hospital Performance, Costs of acute admitted patients in public hospitals in 2011-12. Sydney: National Health Performance Authority; 2015.

6. Australian Orthopaedic Association National Joint Replacement Registry. Hip, knee and shoulder arthroplasty - 2022 annual report. Adelaide: Australian Orthopaedic Association; 2022.
